# P-1766. Evaluation of a Comprehensive Antimicrobial Stewardship Initiative for Gram-Negative Bacteremia in 21 Acute Care Hospitals

**DOI:** 10.1093/ofid/ofae631.1929

**Published:** 2025-01-29

**Authors:** Tara M Rogers, Michael J Trisler, Rachael Ours, Aaron Pickering, William N Stewart, Joseph Welch, Heather Salata, Mary-Teresa Heffern, Megan Wein, Cybele Abad, Amber A Oakes, Howard J Ergen, Jasanjeet Jawanda, Alex Trzebucki, Sunish Shah, Sahil Angelo, Tina Khadem, Justin Ludwig, Chelsea Woodworth, Stephanie Shealy May, John J Veillette, Ryan K Shields, Erin K McCreary, J Ryan Bariola

**Affiliations:** UPMC, Pittsburgh, Pennsylvania; UPMC Presbyterian Shadyside, Pittsburgh, Pennsylvania; UPMC, Pittsburgh, Pennsylvania; UPMC St. Margaret, Pittsburgh, Pennsylvania; UPMC, Pittsburgh, Pennsylvania; UPMC Hamot, Erie, Pennsylvania; UPMC, Pittsburgh, Pennsylvania; UPMC, Pittsburgh, Pennsylvania; UPMC Mercy, Pittsburgh, Pennsylvania; UPMC, Pittsburgh, Pennsylvania; UPMC Magee-Womens Hospital, Pittsburgh, Pennsylvania; UPMC Jameson, Jamestown, Pennsylvania; University of Pittsburgh Medical Center, Vincennes, Indiana; Department of Medicine / University of Pittsburgh School of Medicine, Pittsburgh, PA; Antibiotic Management Program, UPMC Presbyterian Hospital, Pittsburgh, PA, Pittsburgh, Pennsylvania; University of Pittsburgh Medical Center, Vincennes, Indiana; UPMC, Pittsburgh, Pennsylvania; Office of Quality and Clinical Research Innovation, University of Pittsburgh Medical Center, Pittsburgh, PA, USA, Pittsburgh, Pennsylvania; University of Pittsburgh Medical Center, Pittsburgh, PA, USA, Pittsburgh, Pennsylvania; Intermountain Healthcare, Salt Lake City, UT; Intermountain Healthcare, Salt Lake City, UT; University of Pittsburgh, Pittsburgh, Pennsylvania; University of Pittsburgh Medical Center, Vincennes, Indiana; UPMC, Pittsburgh, Pennsylvania

## Abstract

**Background:**

Shorter antibiotic courses are non-inferior to longer for treatment of uncomplicated gram-negative bacteremia (GNB). This study evaluated the impact of a pharmacist-driven stewardship effort to use clinical decision support software (CDSS; ILÚM Insight, Infectious Diseases Connect, Inc.) plus an evidence-based treatment algorithm on antibiotic duration of therapy (DOT) for GNB in the UPMC health system, USA.

**Figure:**
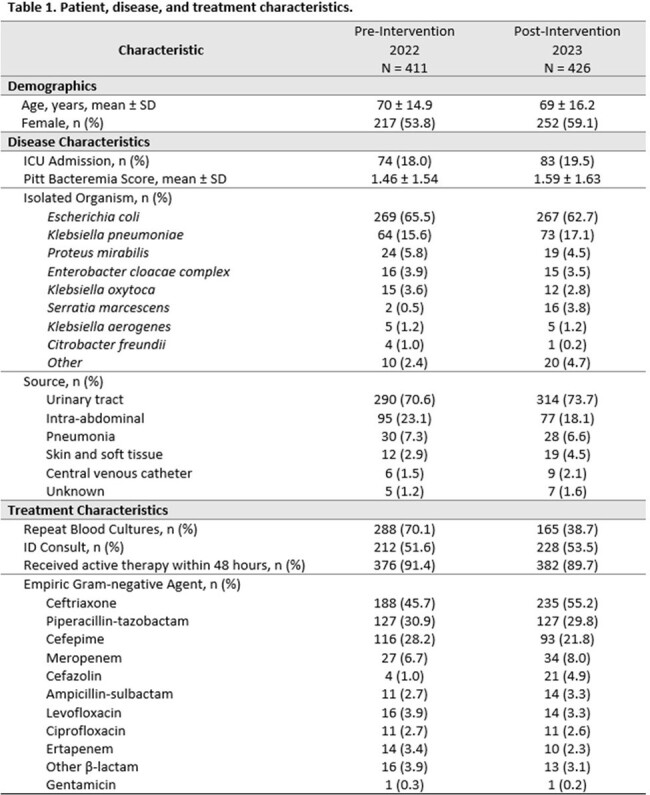

**Methods:**

This retrospective cohort study utilized CDSS alerts for Enterobacterales GNB in adults from March to August 2022 (pre-intervention) and March to August 2023 (post-intervention). We excluded patients with complicated or polymicrobial GNB, admission ≤ 24 hours or death ≤ 72 hours after blood culture result, index culture outside of a UPMC facility, or severe immunocompromising condition.

We matched patients using a propensity score incorporating age, sex, and comorbidities. We compared total antibiotic DOT using a Wilcoxon rank-sum test, and compared key secondary outcomes, including infection-related readmission, *C. difficile* infection (CDI), development of multi-drug resistant organisms (MDRO), and all-cause death using Cochran-Mantel-Haenszel statistics.

**Figure:**
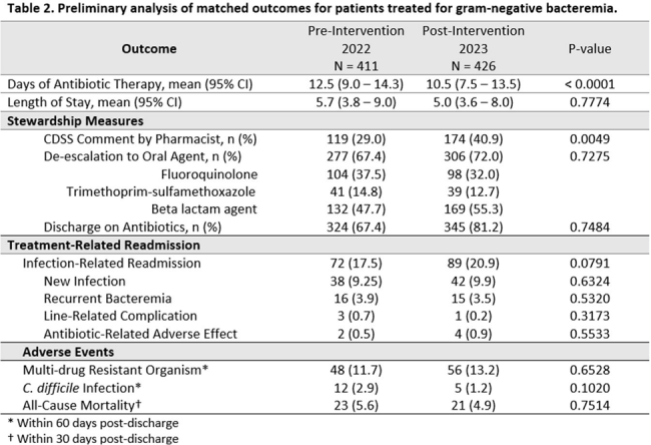

**Results:**

After initial screening, 1586 patients met inclusion criteria; of these, 411 and 426 patients were included in final pre- and post-intervention analyses, respectively (Table 1). Primary reasons for exclusion were complicated (35.8%) or polymicrobial (7.3%) infection. Median DOT was shorter post-intervention (10.5 days) versus pre-intervention (12.5 days; p < 0.0001), and patients receiving ≤ 7 days of therapy increased from 14.8% to 19.6% post-intervention. Pharmacist engagement with CDSS alerts increased from 29% to 41% (p < 0.005). Rate of IV to oral de-escalation, percent discharge on antibiotics, length of stay, and rate of treatment-emergent adverse effects did not differ significantly pre- and post-intervention (Table 2).

**Conclusion:**

Preliminary findings suggest that pharmacist engagement with CDSS alerts in combination with an evidence-based treatment algorithm is associated with shorter antibiotic therapy duration. Ongoing subgroup analyses will help target future stewardship efforts to certain patient populations or facilities for continued improvement.

**Disclosures:**

**Ryan K. Shields, PharmD, MS**, Allergan: Advisor/Consultant|Cidara: Advisor/Consultant|Entasis: Advisor/Consultant|GSK: Advisor/Consultant|Melinta: Advisor/Consultant|Melinta: Grant/Research Support|Menarini: Advisor/Consultant|Merck: Advisor/Consultant|Merck: Grant/Research Support|Pfizer: Advisor/Consultant|Roche: Grant/Research Support|Shionogi: Advisor/Consultant|Shionogi: Grant/Research Support|Utility: Advisor/Consultant|Venatorx: Advisor/Consultant|Venatorx: Grant/Research Support **Erin K. McCreary, PharmD**, Abbvie: Advisor/Consultant|Basilea: Advisor/Consultant|Ciadara: Advisor/Consultant|Entasis: Advisor/Consultant|Ferring: Advisor/Consultant|GSK: Advisor/Consultant|GSK: Honoraria|Melinta: Advisor/Consultant|Merck: Advisor/Consultant|Pfizer: Honoraria|Shionogi: Advisor/Consultant|Shionogi: Honoraria

